# Cerebrospinal fluid penetration of meropenem in neurocritical care patients with proven or suspected ventriculitis: a prospective observational study

**DOI:** 10.1186/s13054-016-1523-y

**Published:** 2016-10-24

**Authors:** Ute Blassmann, Anka C. Roehr, Otto R. Frey, Cornelia Vetter-Kerkhoff, Niklas Thon, William Hope, Josef Briegel, Volker Huge

**Affiliations:** 1 Department of Pharmacy, University Hospital of Munich, Marchioninistrasse 15, Munich, 81377 Germany; 2 Department of Pharmacy, Heidenheim General Hospital, Schlosshausstrasse 100, Heidenheim, 89522 Germany; 3 Department of Neurosurgery, University Hospital of Munich, Marchioninistrasse 15, Munich, 81377 Germany; 4 Department of Molecular and Clinical Pharmacology, University of Liverpool, Sherrington Building, Liverpool, L69 3GE UK; 5 Department of Anaesthesiology, University Hospital of Munich, Marchioninistrasse 15, Munich, 81377 Germany

**Keywords:** Meropenem, Cerebrospinal fluid, Pharmacokinetics, Ventriculitis, Neurocritical care patients

## Abstract

**Background:**

Ventriculitis is a complication of temporary intraventricular drains. The limited penetration of meropenem into the cerebrospinal fluid (CSF) is well known. However, ventricular CSF pharmacokinetic data in patients with ventriculitis are lacking. The aim of this study was to evaluate meropenem pharmacokinetics in the serum and CSF of neurocritical care patients with proven or suspected ventriculitis.

**Methods:**

We conducted an observational pharmacokinetic study of neurocritical care patients with proven or suspected ventriculitis receiving meropenem. Multiple blood and CSF samples were taken and were described using nonparametric pharmacokinetic modelling with Pmetrics.

**Results:**

In total, 21 patients (median age 52 years, median weight 76 kg) were included. The median (range) of peak and trough concentrations in serum were 20.16 (4.40–69.00) mg/L and 2.54 (0.00–31.40) mg/L, respectively. The corresponding peak and trough concentrations in CSF were 1.20 (0.00–6.20) mg/L and 1.28 (0.00–4.10) mg/L, respectively, with a median CSF/serum ratio (range) of 0.09 (0.03–0.16). Median creatinine clearance ranged from 60.7 to 217.6 ml/minute (median 122.5 ml/minute). A three-compartment linear population pharmacokinetic model was most appropriate. No covariate relationships could be supported for any of the model parameters. Meropenem demonstrated poor penetration into CSF, with a median CSF/serum ratio of 9 % and high interindividual pharmacokinetic variability.

**Conclusions:**

Administration of higher-than-standard doses of meropenem and therapeutic drug monitoring in both serum and CSF should be considered to individualise meropenem dosing in neurocritical care patients with ventriculitis.

## Background

Neurocritical care patients often require implantation of an intraventricular catheter (IVC) to manage hydrocephalus and monitor intracranial pressure [1, 2]. IVC-related ventriculitis and/or meningitis are the primary complications in these patients [3]. Infection rates are approximately 10 %, and they are associated with significant morbidity and mortality [1, 3]. Meropenem plus vancomycin is a frequently used antimicrobial combination for management of IVC-related infections because of its broad spectrum of antimicrobial activity [2, 4]. Nevertheless, relatively little is known about the pharmacokinetics (PK) of meropenem in the cerebrospinal fluid (CSF) of patients with ventriculitis [5, 6].

Meropenem exhibits time-dependent antimicrobial activity [7]. Its antibacterial effect is related primarily to the fraction of the dosing interval that the unbound concentration is above the minimum inhibitory concentration (*f*T_>MIC_) [7]. The bactericidal activity of meropenem in laboratory animal models requires 40–50 % *f*T_>MIC_ in plasma [8, 9]. The relevance of this estimate for infections within the central nervous system (CNS) is not known. A significant challenge for critical care physicians is achieving and maintaining appropriate concentrations at the target site of infection (i.e., the CSF for neurocritical care patients). In randomised clinical trials, meropenem was as effective as cefotaxime and ceftriaxone for treating community-acquired bacterial meningitis in children and adults [10, 11]. The penetration of antibiotics into the CNS is dependent on several factors, such as the presence of meningeal inflammation [5, 6]. The meninges in ventriculitis are typically normal or only minimally inflamed [5, 6]. Thus, penetration into the CNS in patients with ventriculitis should not be extrapolated from other patient populations. While meropenem is recommended for the empirical treatment of meningitis and IVC-related infections [2, 4], there are no comparative efficacy trials for patients with minimally inflamed meninges with ventriculitis and no clear idea of optimal regimens for this patient group.

The aim of this study was to evaluate meropenem concentrations in the serum and CSF of neurocritical care patients with IVC and proven or suspected ventriculitis. This study provides a first critical step in identifying regimens of meropenem that can be used to treat patients with ventriculitis. These regimens can then be further studied in clinical trials and are a way in which clinical outcomes can potentially be improved.

## Methods

### Study design and population

This prospective, observational PK study was performed at the intensive care unit (ICU) of Munich University Hospital, Munich, Germany, between April 2014 and January 2016. The trial was conducted in accordance with the Declaration of Helsinki. Ethical approval was obtained from the university ethics committee (registration number 111-14). Written informed consent was obtained from all patients or their legally authorised representatives before enrolment. Patients were enrolled in the study if they were admitted to the ICU having an IVC and proven or suspected ventriculitis. Proven ventriculitis was defined as a positive CSF culture combined with clinical signs of infection [12]. Suspected ventriculitis was defined by abnormal CSF parameters, such as low CSF glucose levels (<50 % of serum glucose), high CSF protein (>50 mg/dl) or CSF pleocytosis, combined with clinical signs of infection and in the absence of a positive CSF culture [12]. Patients were excluded if they were under 18 years of age or death within 72 h was predicted.

### Drug administration

Meropenem (Meropenem Hikma®; Hikma Pharma, Gräfelfing, Germany) was administered as a prolonged infusion over 4 h using a syringe pump. The dose was 2000 mg every 8 h for all patients, except for those with adverse drug effects or renal impairment (creatinine clearance [CrCL] ≤50 ml/minute), for whom the dose was reduced to 1000 mg every 8 h at the discretion of the attending physician.

### Study procedures

Serial blood and CSF sampling occurred for initial dose and steady state (daily on days 1–3, followed by every second or third day). Blood samples (4 ml) were collected using the indwelling arterial catheter just before the start of the infusion (serum trough concentration [C_min_]) and after the end of the infusion (serum peak concentration [C_max_]). CSF samples (1 ml) were collected using the indwelling IVC nearest to the site of insertion (3-ml volume to sampling location) simultaneously with each blood sample just before the start of the infusion (cerebrospinal fluid concentration at serum trough concentration [C_trough_]) and after the end of the infusion (cerebrospinal fluid concentration 4 h after serum trough concentration [C_after 4h_]). Samples were centrifuged for 5 minutes at 4000 rpm immediately after sample collection and aliquoted into 2-ml propylene tubes (Eppendorf, Hamburg, Germany). Aliquots were stored at −80 °C within 45 minutes after sample collection for a maximum of 4 weeks until assay. Additional data were obtained from the medical record, including weight, height, serum creatinine, bilirubin, serum C-reactive protein (CRP), serum interleukin-6 (IL-6), serum procalcitonin (PCT), serum leucocytes, CSF cells, CSF erythrocytes, CSF IL-6, CSF glucose, CSF protein, CSF drain in 24 h, Simplified Acute Physiology Score II (SAPS II), Sepsis-related Organ Failure Assessment (SOFA) score, Glasgow Coma Scale (GCS) and dexamethasone therapy.

### Bioanalytical methodology

Serum and CSF concentrations of meropenem were analysed using a validated high-performance liquid chromatography assay with ultraviolet detection. The analyses were performed in the laboratory of the pharmacy department of Heidenheim General Hospital [13]. The assay was linear from 1 to 30 mg/L in serum and from 0.5 to 5 mg/L in CSF with a relative SD for intra- and interday precision and accuracy <5 % at high, medium and low concentrations. The limits of quantification were 0.5 mg/L for serum samples and 0.2 mg/L for CSF samples.

### Population pharmacokinetic analysis

The oncentration–time data for meropenem in serum and CSF were analysed using a non-parametric population methodology with the nonparametric adaptive grid program Pmetrics version 1.3.2 [14]. The structure of the PK mathematical model fitted to the study data was modified from a previously published meropenem model [15] and took the following form:

1 $$ \mathrm{d}\mathrm{X}1/\mathrm{d}\mathrm{t}=\kern0.5em \mathrm{R}\left(\mathrm{t}\right){\textstyle \hbox{-}}\mathrm{C}\mathrm{L}/{\mathrm{V}}_{\mathrm{c}}\ast \mathrm{X}1{\textstyle \hbox{-} }{\mathrm{k}}_{\mathrm{c}\mathrm{p}}\ast \mathrm{X}1{\textstyle \hbox{-} }{\mathrm{k}}_{\mathrm{bc}}\ast \mathrm{X}1+{\mathrm{k}}_{\mathrm{pc}}\ast \mathrm{X}2+{\mathrm{k}}_{\mathrm{bc}}\ast \mathrm{X}3 $$

2 $$ \mathrm{d}\mathrm{X}2/\mathrm{d}\mathrm{t}={\mathrm{k}}_{\mathrm{cp}}\ast \mathrm{X}(1){\textstyle \hbox{-} }{\mathrm{k}}_{\mathrm{pc}}\ast \mathrm{X}2 $$

3 $$ \mathrm{d}\mathrm{X}3/\mathrm{d}\mathrm{t}={\mathrm{k}}_{\mathrm{cb}}\ast \mathrm{X}(1){\textstyle \hbox{-} }{\mathrm{k}}_{\mathrm{bc}}\ast \mathrm{X}3 $$

These three equations describe a three-compartment pharmacokinetic model with central, peripheral and CSF compartments denoted by the numbers 1, 2 and 3, respectively. R(t) in milligrams per hour represents the zero-order infusion of meropenem. Meropenem was cleared from the central compartment (clearance in litres per hour), which also has a volume (V_c_; given in litres). *K*_cp_, *K*_pc_, *K*_cb_ and *K*_bc_ represent first-order transfer constants connecting the various compartments. The CSF compartment (X3) has an apparent CSF volume (V_CSF_; given in litres). Equation (1) describes the rate of change of the amount of meropenem (in milligrams) in the central compartment (X1). Equation (2) describes the rate of change of the amount of meropenem (in milligrams) in the peripheral compartment (X2). Equation (3) describes the rate of change of the amount of meropenem (in milligrams) in the CSF compartment (X3).

In Pmetrics, error can be separately attributed to assay variance and additional process noise such as errors in sampling time or dosing. The data were weighted using the inverse of the estimated assay variance. Additional process noise such as errors in sampling time or dosing was modelled using a fixed lambda as an additive error term in Pmetrics.

### Population pharmacokinetic model diagnostics

The fit of the PK model to the data set was assessed in the following ways: (1) the log-likelihood value, (2) the coefficient of determination (*r* ^2^) of the linear regression and (3) visual inspection of diagnostic scatterplots, where model predictions were generated either by the median population parameter values or by the medians of each subject’s individual Bayesian posterior parameter value distributions.

### Population pharmacokinetic covariate screening

The impact of weight, CrCL, bilirubin, serum CRP, serum IL-6, serum PCT, serum leucocytes, CSF cells, CSF erythrocytes, CSF IL-6, CSF glucose, CSF protein, CSF drain in 24 h, SAPS II, SOFA score and GCS as covariates was initially assessed by visual inspection. For that reason, graphical representation in Pmetrics of each covariate versus population parameter was performed to evaluate for inclusion in the final model.

### Other pharmacokinetic calculations

C_max_ and C_min_ in serum and C_after 4 h_ and C_troug﻿h_ in﻿ CSF are the observed values. The average AUC for each patient was calculated using the Bayesian posterior parametric estimates from the final model using the trapezoidal rule in Pmetrics. We divided each subject’s cumulative AUC (AUCf) by the total time in hours and multiplied the result by 24 to estimate the daily average AUC (AUC_0–24_). Penetration of meropenem into CSF was described using the CSF/serum ratio, which was calculated by dividing the CSF AUCf by the serum AUCf. Half-life was calculated using transfer rate constants. CrCL was calculated using the Cockcroft-Gault equation [16]. All calculations were performed using IBM SPSS Statistics version 23.0 software (IBM, Armonk, NY, USA).

### Assessment of meropenem concentration in CSF

Simulations of 1000 patients were performed using Pmetrics to compare different dosing regimens in this study population (2000 mg every 8 h, 4000 mg every 8 h, 4000 mg every 6 h, 5000 mg every 6 h; 4-h infusion). In addition, probability of target attainment (PTA) in CSF was analysed using Pmetrics to achieve meropenem concentrations in CSF of 1 mg/L, 2 mg/L and 4 mg/L. Linear regression was performed using Pmetrics. PTA presentation was performed using IBM SPSS software.

## Results

In total, 209 blood samples and 199 CSF samples from 21 patients were included in the model. The demographic and general clinical characteristics of patients are shown in Table 1. Briefly, the study population was relatively young (median age 52 years, range 46–80 years) and had well-preserved renal function on the day of inclusion (median CrCL 120.1 ml/minute, range 52.3–217.6 ml/minute). The median (range) SAPS II score was 47 (13–62). All patients received vancomycin therapy in addition to meropenem. Vancomycin was replaced by linezolid in one patient (4.8 %), owing to an increase in serum creatinine level. Seven patients (33.3 %) received concomitant fosfomycin for 7 days, although one (4.8 %) of them also received rifampicin, which then was replaced by fosfomycin. Patient 1 additionally received dexamethasone during the first 2 days, and patient 14 additionally received dexamethasone during the first 5 days. The most frequent neurological disease was subarachnoid haemorrhage, observed in 17 (81.0 %) patients. In the remaining four patients, an IVC was placed for intracranial bleeding (4.8 %), tumour (9.5 %) or traumatic brain injury (4.8 %). A total of 20 patients (95.2 %) were CSF culture-negative, and one patient (4.8 %) had a positive culture for *Pseudomonas aeruginosa* that was susceptible to meropenem.

**Table 1:** Patient characteristics

Characteristic	Data
Age, years, median (range)	52 (46–80)
Weight, kg, median (range)	76 (55–105)
Body mass index, kg/m^2^, median (range)	25.95 (20–33)
Sex, male/female	52.4 %/47.6 %
CrCL on day of inclusion, ml/minute, median (range)	120.1 (52.3–217.6)
CRP in serum on day of inclusion, mg/dl, median (range)	3.1 (0.4–36.7)
Interleukin-6 in serum on day of inclusion, pg/ml, median (range)	13.7 (2.4–274.0)
CSF drain in 24 h on day of inclusion, median (range)	183 (21–360)
Interleukin-6 in CSF on day of inclusion, pg/ml, median (range)	3398 (140–24,522)
Cells in CSF on day of inclusion, *n*/μl, median (range)	503 (4–2894)
Protein in CSF on day of inclusion, mg/L, median (range)	107 (13–303)
Glucose in CSF on day of inclusion, mg/dl, median (range)	72 (47–126)
Glucose CSF/serum ratio on day of inclusion, median (range)	0.55 (0.38–0.99)
SAPS II on day of inclusion	47 (13–62)
SAPS II on day of exclusion, median (range)	32 (13–61)
SOFA score on day of inclusion, median (range)	6 (1–12)
SOFA score on day of exclusion, median (range)	2.5 (0–8)
30-day mortality	0

*Abbreviations: CrCL* Estimated creatinine clearance (calculated using the Cockcroft-Gault equation [16]), *CRP* C-reactive protein, *CSF* Cerebrospinal fluid, *SOFA* Sepsis-related Organ Failure Assessment, *SAPS II* Simplified Acute Physiology Score II

In serum, the median C_max_ (range) was 20.16 (4.40–69.00) mg/L and the median C_min_ (range) was 2.54 (0.00–31.40) mg/L. In CSF, the median C_after 4h_ (range) was 1.20 (0.00–6.20) mg/L and the median C_trough_ (range) was 1.28 (0.00–4.10) mg/L. The median CrCL ranged from 60.7 to 217.6 ml/minute (median 122.5 ml/minute). Individual observed meropenem concentrations and median CrCL values are shown in Table 2. The median AUC_0–24_ in CSF was 26.56 mg∙h/L, and in serum it was 350.22 mg∙h/L. The values for the AUC_0–24_ in CSF and serum ranged from 7.44 to 85.53 mg∙h/L and from 112.95 to 768.63 mg∙h/L, respectively. The median CSF/serum ratio (range) was 0.09 (0.03–0.16). Individual AUC_0–24_ and penetration results are shown in Table 3.

**Table 2:** Observed meropenem concentrations in serum and cerebrospinal fluid

		Meropenem dosing 1000 mg				Meropenem dosing 2000 mg			
Patient number	CrCL (ml/minute)	C_min_ (mg/L)	C_max_ (mg/L)	C_trough_ (mg/L)	C_after 4h_ (mg/L)	C_min_ (mg/L)	C_max_ (mg/L)	C_trough_ (mg/L)	C_after 4h_ (mg/L)
1	60.7	3.80	N/A	2.00	N/A	10.90	48.60	3.00	4.00
2	156.8	6.90	11.00	N/A	N/A	4.60	26.35	2.75	2.60
3	83.8	4.80	24.10	2.60	1.90	13.80	45.30	2.25	3.45
4	162.9	N/A	N/A	N/A	N/A	1.10	16.40	0.60	0.60
5	217.6	<0.5	5.80	N/A	N/A	2.90	19.40	0.31	0.53
6	124.5	N/A	N/A	N/A	N/A	0.86	19.20	0.76	0.86
7	120.1	N/A	N/A	N/A	N/A	<0.5	15.20	0.43	0.78
8	73.1	N/A	N/A	N/A	N/A	11.00	64.80	3.35	3.00
9	92.1	0.81	12.30	1.08	0.97	3.05	19.65	1.40	1.97
10	96.3	N/A	N/A	N/A	N/A	1.31	33.30	1.62	1.04
11	142.9	N/A	12.82	N/A	1.14	1.02	19.33	0.62	0.74
12	174.1	0,62	5.62	0.39	0.32	N/A	N/A	N/A	N/A
13	100.3	1.38	10.65	1.47	1.15	0.79	19.80	0.87	0.80
14	96.2	N/A	N/A	N/A	N/A	9.83	32.94	2.27	2.19
15	108.8	N/A	N/A	N/A	N/A	1.13	17.51	1.18	0.88
16	125.0	N/A	N/A	N/A	N/A	2.99	27.01	0.92	1.17
17	144.7	N/A	N/A	N/A	N/A	2.44	18.15	0.74	0.58
18	90.5	N/A	20.10	N/A	2.28	7.63	33.80	2.39	1.84
19	131.3	2.50	17.47	0.89	0.83	6.22	26.94	1.79	1.82
20	174.6	N/A	N/A	N/A	N/A	2.63	23.23	1.52	1.58
21	122.5	1.69	10.57	<0.2	0.25	3.65	16.74	0.31	0.44
Median	122.5	1.69	11.65	1.08	1.05	2.95	21.51	1.29	1.10
Minimum	60.7	<0.5	4.40	<0.2	<0.2	<0.5	10.70	<0.2	0.24
Maximum	217.6	7.10	26.60	3.10	2.80	31.40	69.00	4.10	6.20

*Abbreviations: C*_*min*_ Median observed serum trough concentration, *C*_*max*_ Median observed serum peak concentration, *C*_*trough*_ Median observed cerebrospinal fluid concentration at serum trough concentration, *C*_*after 4h*_ Median observed cerebrospinal fluid concentration 4 h after serum trough concentration, *N/A* Not available (patient with only meropenem 1000 mg or only 2000 mg intravenously every 8 h), *CrCL* Estimated creatinine clearance (calculated using the Cockcroft-Gault equation [16])

Observed meropenem concentrations after 1000 mg or 2000 mg intravenously every 8 h (4-h infusion)

**Table 3:** Pharmacokinetic properties of meropenem in serum and cerebrospinal fluid

Patient number	CL (L/h)	V_c_ (L)	*t*_1/2_ (h)	AUC_0–24_ serum (mg∙h/L)	AUC_0–24_ CSF (mg∙h/L)	CSF/serum ratio	*t*_1/2cb_ (h)	*t*_1/2bc_ (h)
1	7.63	6.45	0.59	768.63	85.53	0.11	3.30	4.62
2	17.88	14.45	0.56	327.09	53.00	0.16	17.33	9.90
3	8.62	14.95	1.20	428.94	48.59	0.11	4.62	3.47
4	20.38	5.05	0.17	300.14	19.37	0.06	13.86	17.33
5	22.87	14.95	0.45	237.60	8.93	0.04	17.33	2.57
6	14.89	5.05	0.24	401.45	18.63	0.05	34.66	69.31
7	23.37	14.95	0.44	258.79	15.02	0.06	34.66	5.78
8	7.88	5.05	0.44	760.20	79.10	0.10	8.66	9.90
9	17.13	14.95	0.60	280.81	26.56	0.09	23.10	17.33
10	11.88	13.75	0.80	489.44	27.25	0.06	69.31	34.66
11	20.63	12.75	0.43	287.15	15.59	0.05	34.66	17.33
12	29.87	9.95	0.23	112.95	10.56	0.09	11.55	9.90
13	15.07	5.05	0.23	234.65	22.10	0.09	23.10	69.31
14	9.63	14.95	1.08	569.59	48.90	0.09	13.86	5.78
15	14.88	5.05	0.24	413.23	25.62	0.06	34.66	69.31
16	17.13	14.95	0.60	337.21	30.99	0.09	23.10	17.33
17	14.98	5.08	0.24	415.22	17.31	0.04	34.66	69.31
18	10.63	5.05	0.33	563.84	42.95	0.08	5.78	8.66
19	13.12	14.95	0.79	394.45	33.99	0.09	34.66	13.86
20	17.13	14.95	0.60	350.22	33.71	0.10	23.10	17.33
21	18.88	14.15	0.52	242.60	7.44	0.03	17.33	1.73
Median	15.07	13.75	0.63	350.22	26.56	0.09	23.10	13.86
Minimum	7.63	5.05	0.17	112.95	7.44	0.03	3.30	1.73
Maximum	29.87	14.95	1.20	768.63	85.53	0.16	69.31	69.31

*Abbreviations: CSF* Cerebrospinal fluid, *CL* Median clearance, *t*_*1/2*_ Median half-life, *V*_*c*_ Median volume of distribution of the central compartment, *AUC*_*0–24*_ Daily average area under the curve, *CSF/serum ratio* Cerebrospinal fluid penetration, *t*_*1/2cb*_ Median absorption half-life into cerebrospinal fluid, *t*_*1/2bc*_ Median elimination half-life of cerebrospinal fluid

Individual pharmacokinetic results in serum and CSF obtained using Pmetrics

### Pharmacokinetic model building

**Fig. 1:**
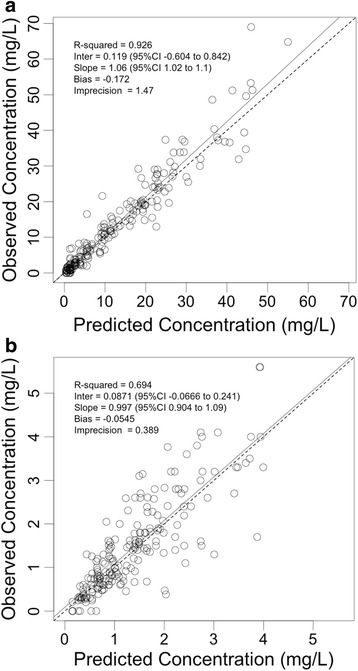
Diagnostic plots for the final population pharmacokinetics model. Individual predicted meropenem concentrations in serum versus observed serum concentrations (*r* ^2^ = 0.926) (**a**) and individual predicted meropenem concentrations in cerebrospinal fluid (CSF) versus observed CSF concentrations (*r* ^2^ = 0.694) (**b**), indicating that 92.6 % in serum and 69.4 % in CSF of the observed variability in meropenem concentrations were explained by the parametric distributions in the model. The *solid black line* shows the linear regression line of fit. The estimates of bias and imprecisions were also acceptable (−0.172 and 1.47 in serum and −0.0545 and 0.389 in CSF, respectively)

The three-compartment model was adequately able to describe the observed concentrations for the full data set. The fit of the population PK model was acceptable according to visual inspection of the observed-versus-predicted plots and *r* ^2^ of the observed-versus-predicted values (*r* ^2^ = 0.926 in serum, *r* ^2^ = 0.694 in CSF) (Fig. 1). Individual PK results in serum and CSF obtained by Pmetrics for the PK model are shown in Table 3. The mean, median and SD for the population parameters identified by Pmetrics for the PK model are shown in Table 4. No covariate relationships could be supported for any of the model parameters.

**Table 4:** Population pharmacokinetic mean, median and SD parameters of meropenem obtained using Pmetrics

	Mean	Median	SD
CL, L/h	16.045	15.025	±5.575
V_c_, L	10.949	13.736	±4.491
*K*_cp_, L^−1^	1.562	1.248	±1.031
*K*_pc_, L^−1^	1.686	1.898	±1.206
*K*_cb_, L^−1^	0.052	0.026	±0.049
*K*_bc_, L^−1^	0.092	0.054	±0.097
V_CSF_, L	82.932	93.902	±18.802

*Abbreviations: CL* Clearance, *V*_*c*_ Volume of distribution of the central compartment, *V*_*CSF*_ Volume of distribution of the cerebrospinal fluid compartment, *K*_cp_, *K*_pc_, *K*_bc_, *K*_cb_ Linear transfer rate constants

### Assessment of meropenem concentration in CSF

**Fig. 2:**
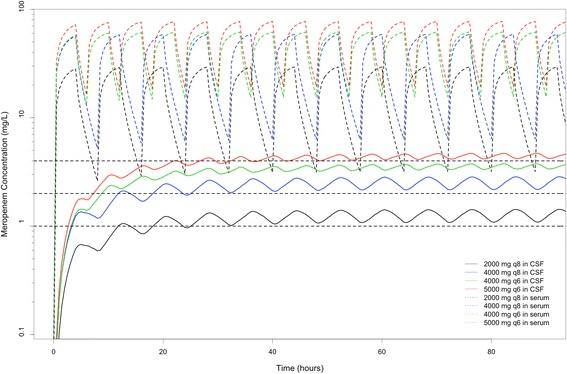
Comparison of different dosing regimens as prolonged infusions over 4 h using the pharmacokinetics model. Median time course of meropenem concentrations simulated in serum and cerebrospinal fluid (CSF) over 4 days. Targeted meropenem trough concentrations in CSF were 1 mg/L, 2 mg/L and 4 mg/L

**Fig. 3:**
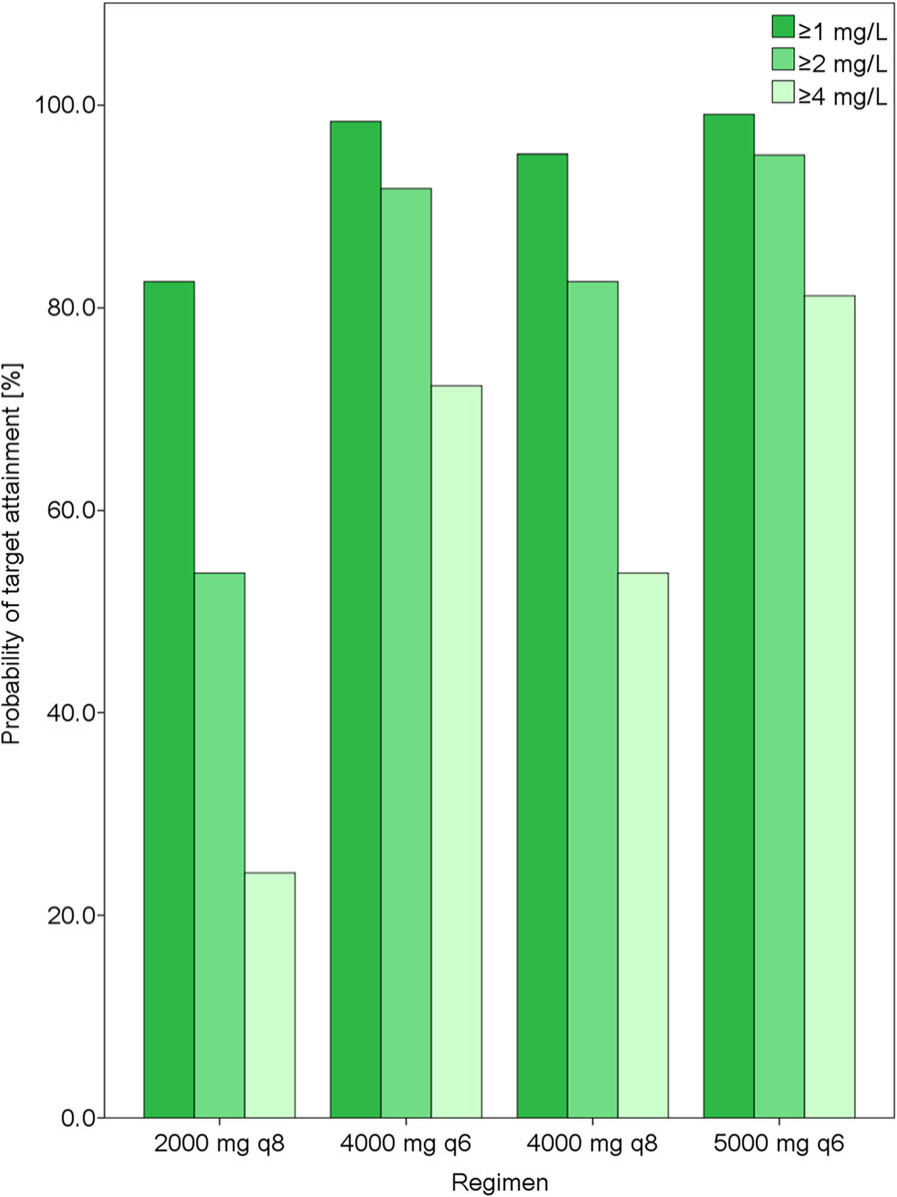
Probability of target attainment in cerebrospinal fluid (CSF) for different dosing regimens as prolonged infusions over 4 h. The proportions of simulated patients who exceeded meropenem trough concentrations in CSF greater than or equal to 1 mg/L, 2 mg/L and 4 mg/L for each regimen are shown

Simulated meropenem concentration–time profiles in serum and CSF of each regimen are shown in Fig. 2. The proportions of simulated patients who exceeded targeted meropenem concentrations in CSF of each regimen are shown in Fig. 3.

## Discussion

To our knowledge, this is the first population PK study of meropenem concentrations in serum and CSF in neurocritical care patients with ventriculitis. Furthermore, it is also the largest study investigating the penetration of meropenem into CSF. We found that meropenem poorly penetrated into CSF, with a median penetration ratio of only 9 %. However, there was considerable interindividual variability in serum and CSF concentrations and resultant CSF/serum ratios. This variation is most likely due to the range of sickness severity and the consequent effect of altered physiology on meropenem exposure, as well as to the integrity of the blood–CSF barrier. These findings are concordant with those derived from previous studies in which researchers have also described large interindividual variability in meropenem concentrations in serum [17] and CSF [18]. However, PK variability was not explained by any covariates. Therefore, our study suggests the need for therapeutic drug monitoring of meropenem in both serum and CSF to avoid treatment failures due to underexposure or overdosing resulting in potential side effects, as suggested by a case report by Lonsdale et al*.* [19].

CSF is produced by the choroid plexus [20]. Drug penetration into CSF is indicative of the transport across the choroid plexus at the blood–CSF barrier [21]. The blood–CSF barrier is ‘leaky’ compared with the blood–brain barrier, and molecules enter the CSF by diffusion at a rate that is inversely proportional to their molecular weight [20, 21]. Our PK model suggests that meropenem penetration (median 23 h) is slower than CSF clearance (median 14 h). Approximately 24–48 h are required to achieve steady-state concentrations in the CSF (Fig. 1). In future studies, researchers could examine innovative ways to achieve effective CSF concentrations as quickly as possible. Robust estimates of early penetration would require optimal sampling in this early treatment period. Neither accumulation of drug nor significant intraindividual variability over the treatment course was observed. The apparently high volume of CSF reflects the relatively low CSF concentrations compared with serum. V_CSF_ should not be viewed as the physiological CSF volume; it is merely a scalar that explains the concentration observed in the CSF.

A CSF penetration of meropenem of 20 % in normal or mildly infected meninges and 39 % in inflamed meninges is described elsewhere [6]. However, studies citing relatively higher CSF penetration (e.g., 21 % [22], 25 % [23], 39 % [10]) have been conducted in patients with bacterial meningitis [10, 23] or with CSF that was collected by lumbar drainage [22]. Nau et al. [18] observed a CSF penetration of meropenem of 4.6 % (range 1.9–8.9 %) in ten neurocritical care patients with extracerebral infections [18]. This is similar to our findings considering high interindividual variability in CSF/serum ratios. In this study, meropenem clearance in serum (16.0 L/h) was greater than that reported in other PK studies in critically ill patients (7.7–9.4 L/h [24], 9.3 L/h [25], 11.5 L/h [26], 13.6 L/h [27]). However, the patients in our study were generally young and without any measured renal dysfunction on the day of inclusion. Interestingly, in a previous study with healthy volunteers (16.3 L/h [28]), researchers described similar meropenem clearance. The fact that the clearance in our patients was similar to that observed in healthy volunteers may be due to the relatively preserved renal function of our patient population (median CrCL 122 ml/minute) in contrast to the renal function in other studies (mean CrCL 84 ml/minute [24], 78 ml/minute [25], 61 ml/minute [26], 100 ml/minute [27]). Greater than normal CrCL is common in neurocritical care patients [29], which may lead to sub-therapeutic concentrations of time-dependent antibiotics such as β-lactam agents. Augmented renal clearance has previously been shown to be an independent predictor of not achieving the pharmacokinetic/pharmacodynamic (PD) target for meropenem [19, 30, 31] as well as other β-lactams [30–33]. Nevertheless, patients’ renal function was not a covariate in our model. CrCL was the most important predictor for meropenem clearance with impaired renal function, although no correlation was observed between CrCL and meropenem clearance above CrCL of 100 ml/minute [31, 34].

The most dreaded pathogens in nosocomial CNS infection are aerobic Gram-negative pathogens (e.g., *P. aeruginosa*), *Staphylococcus aureus* and *Staphylococcus epidermidis* [2, 4]. From a PD point of view, CSF concentrations in our study population exceeded minimum inhibitory concentrations (MICs) for most members of the *Enterobacteriaceae* family (<0.125 mg/L), including *Klebsiella pneumoniae* and methicillin-sensitive *S. aureus* (0.25 mg/L) [9]. However, only 53.8 % of the simulated patients exceeded CSF trough concentrations of 2 mg/L with 2000 mg meropenem every 8 h, assuming all drug in the CSF is unbound (Fig. 3). In contrast, 95.1 % of simulated patients exceeded CSF trough concentrations of 2 mg/L with a regimen of 5000 mg meropenem every 6 h (Fig. 3). Therefore, in neurocritical care patients with CNS infections caused by pathogens with borderline susceptibility such as *P. aeruginosa* (2 mg/L) [9], the standard dosing regimen of meropenem 2000 mg every 8 h as a prolonged infusion is unlikely to achieve adequate CSF concentrations. More work is required to better understand PD targets at the site of infection for patients with ventriculitis.

There are several limitations of this study. First, the study was relatively small, which may have hampered robust estimates of the extent of PK variability and the identification of covariates that may have explained some of the observed variance. CrCL was estimated because the measurement is not routinely performed in routine clinical care. Second, we measured total drug concentrations because protein binding is not relevant for low to moderately protein-bound drugs (unbound fraction 91–98 %) [9]. Finally, all but one patient had suspected ventriculitis without a positive CSF culture. Therefore, we were unable to establish PK–PD relationships. Such an analysis would have been helpful to help establish drug exposure targets at the site of infection.

## Conclusions

To our knowledge, this is the largest PK study of neurocritical care patients with proven or suspected ventriculitis. We found that meropenem showed relatively low penetration into CSF. Furthermore, high interindividual variability in serum and CSF concentrations was observed. Assuming MIC serum breakpoints, adequate CSF concentrations are not assured for pathogens with borderline susceptibility such as *P. aeruginosa*. To address this challenge, novel dosing strategies should be investigated in further clinical studies with high daily dosages and/or with administration by continuous infusion to avoid antibiotic underexposure in the context of augmented elimination or impaired target side penetration. The safety of these higher-dose regimens must be established. An alternative approach to optimising meropenem exposure is to use individualised dosing to achieve the desired drug exposure in both serum and CSF.

## Key message

- Currently recommended regimens for meropenem for proven or suspected ventriculitis may lead to insufficient drug concentrations in cerebrospinal fluid. Therefore, novel treatment strategies, including the possibility of therapeutic drug monitoring within serum and cerebrospinal fluid, should be investigated in further clinical studies.

## Abbreviations

AUC_0–24_: Daily average area under the curve
AUCf: Cumulative area under the curve
C_after 4h_: Cerebrospinal fluid concentration 4 h after serum trough concentration
CL: Clearance
C_max_: Serum peak concentration
C_min_: Serum trough concentration
CNS: Central nervous system
CrCL: Creatinine clearance
CRP: C-reactive protein
CSF: Cerebrospinal fluid
C_trough_: Cerebrospinal fluid concentration at serum trough concentration
*f*T_>MIC_: Fraction of the dosing interval that the unbound concentration is above the minimum inhibitory concentration
GCS: Glasgow Coma Scale
ICU: Intensive care unit
IL: Interleukin
IVC: Intraventricular catheter
*K*_bc_: Transfer constant from the cerebrospinal fluid compartment
*K*_cb_: Transfer constant to the cerebrospinal fluid compartment
*K*_cp_: Transfer constant to the peripheral compartment
*K*_pc_: Transfer constant from the peripheral compartment
MIC: Minimum inhibitory concentration
N/A: Not available
PCT: Procalcitonin
PD: Pharmacodynamics
PK: Pharmacokinetics
PTA: Probability of target attainment
R(t): Meropenem infusion rate
SAPS II: Simplified Acute Physiology Score II
SOFA: Sepsis-related Organ Failure Assessment
*t*_1/2_: Half-life
*t*_1/2cb_: Absorption half-life into cerebrospinal fluid, *t*_1/2bc_, Elimination half-life of cerebrospinal fluid
V_c_: Volume of distribution of the central compartment
V_CSF_: Volume of d﻿istribution of the cerebrospinal fluid compartment
X1: Central compartment
X2: Peripheral compartment
X3: Cerebrospinal fluid compartment
